# Supplementary figures and images for: Serologic and molecular evidence for circulation of Crimean-Congo hemorrhagic fever virus in ticks and cattle in Zambia
Source: PLoS Negl Trop Dis. 2021 Jun 1;15(6):e0009452. doi: 10.1371/journal.pntd.0009452 (PMC8195391; doi:10.1371/journal.pntd.0009452)

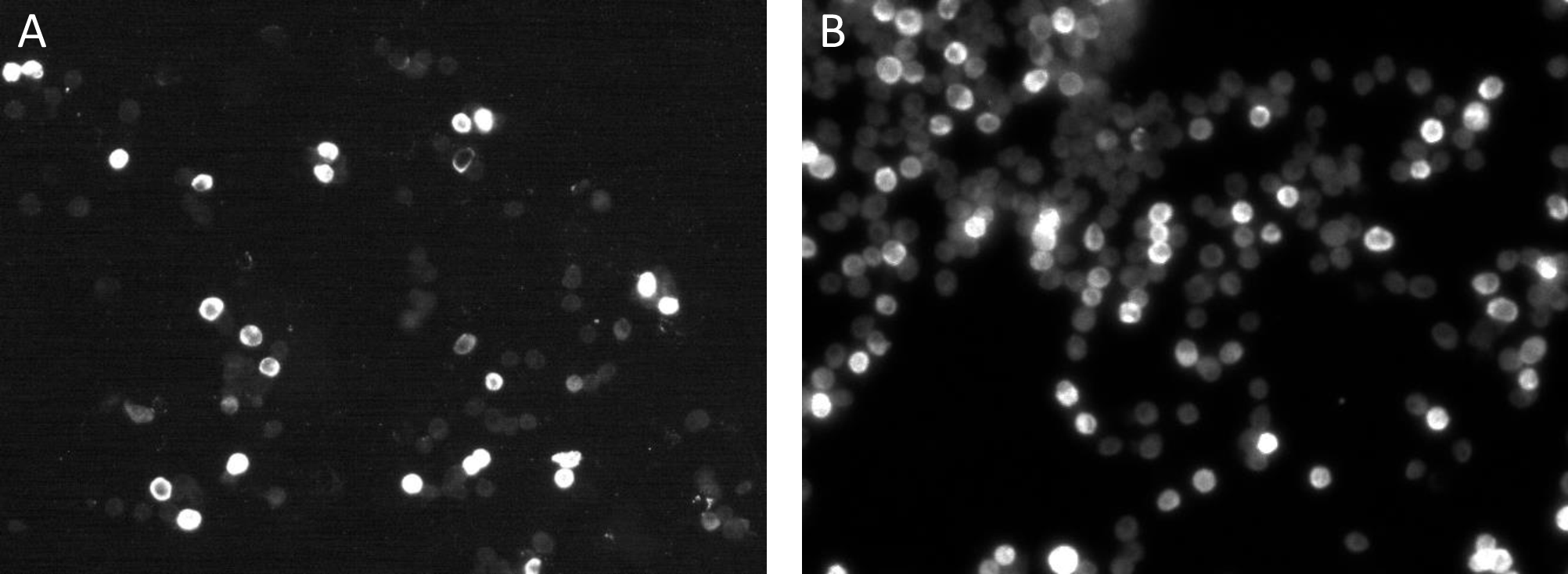

Supplement: S1 Fig — Expression of CCHFV N was confirmed with CCHFV N-immunized rabbit serum (A). Local cattle sera were screened for CCHFV N-specific IgG through an immunofluorescence assay. Typical fluorescence patterns of positive cells with cattle serum are shown (B). (TIF) [file pntd.0009452.s001.tif]
