# Supplementary material for: Serologic and molecular evidence for circulation of Crimean-Congo hemorrhagic fever virus in ticks and cattle in Zambia
Source: PLoS Negl Trop Dis. 2021 Jun 1;15(6):e0009452. doi: 10.1371/journal.pntd.0009452 (PMC8195391; doi:10.1371/journal.pntd.0009452)
Supplement: Attachment — Submitted filename: Response & Rebuttal.docx [file pntd.0009452.s005.docx]

**To Reviewer #1**

**The design of the study was simple and appropriate to address the author’s objective. The methods used in the study were also appropriate. One request from the reviewer is to provide the primer sets (and the target position) for the L gene. Although authors described that they could not detect L gene, it should be helpful to provide that information.**

Thank you for your comment. According to the comment, S2 Table showing primers specific for the L genome segment was attached to the revised manuscript. In addition, description on the attempt for the L genome segment detection was corrected as follows:

“The L segment was not detected by RT-PCR with multiple sets of degenerate primers designed based on conserved sequences in CCHFVs (S2 Table)” (lines 251-253).

**The results presented in this study was shown clearly.**

We really appreciate your comment.

**Conclusion of the study was clear and well supported from the presented data.**

Thank you for your valuable comment.

The reassortant between African and Asian CCHFVs is one of the exciting finding in this manuscript. Reviewer requested to author to discuss a little bit more about the reassortant. Is there any CCHFV reassortment reported of African M segment in Asia? Why only M segment is reassorted in the African CCHFV? Is it possible that L segment is also originated from Asian CCHFV in African CCHFV?

We appreciate your comment. Following sentences with two additional references (31 and 32) were added in the revised manuscript (lines 338-348):

“To our knowledge, CCHFV with the African M segment has never been found outside Africa except for Spain (Negredo et al. 2021 Emerg Infect Dis). In addition, there are no reports of African CCHFV with the Asian-origin S or L segment, whereas the viruses with the African S genome segment have been detected in Russia and United Arab Emirates (Khalafalla et al. 2021 J Gen Virol). The mechanism underlying the high frequency of M segment reassortment compared to the S and L segments is totally unclear. The N and RNA-dependent RNA polymerase encoded by the S and L segments, respectively, might be more important factors than proteins encoded in the M segment to effectively infect endogenous vertebrate or tick hosts, thereby efficiently maintaining viral lifecycle in Africa. However, the CCHF phylogeography has not been comprehensively understood yet due to limited and geographically biased sequence data of the viruses.

Authors reported the circulation of CCHFV in Zambia for the first time. Although authors could not isolate the live virus from their samples, the data shown in here with the serologic data and the genetical data, was enough to support their conclusion. Only few requests were provided from the reviewer above to support their discussion.

We really appreciate your positive comment.

**To Reviewer #2**

The authors describe the first evidence of CCHFV in Zambia based on the detection of IgG antibodies to nucleocapsid protein in 88 cattle sera collected 2012-2015 and the presence of viral RNA in 11 Hyalomma ticks collected 2015-2016. The objective and the public health relevance are clearly described and the manuscript is well written.

Can the authors please provide a statement in Methods confirming that the experimental protocol/protocols for sampling cattle were approved by a licensing committee (name the authorizing body and ethical permit number). If the authors feel that this is not relevant to their study, depending on the regulations in Zambia, can the authors please provide a statement in Methods explaining why there is no need for such a permit.

The authors refer to reference 8 for the cattle samples. The paper referred to describes the collection of 942 samples in 2014. Can the authors please add information on the remaining samples or provide an additional reference describing their origin?

Thank you for your comment. The present study was conducted as a collaborative study with Central Veterinary Research Institute, Ministry of Fisheries and Livestock. Sample collection was approved by the Department of Veterinary Services according to the Animal Health Act No. 27 of 2010. This explanation was added in the revised manuscript (lines 102-105). The samples used in the present study were collected in the previous study (reference #8, Saasa et al. 2018) and a follow-up study in 2015. In order to more accurately explain the samples used in this study, the sentence was modified as follows:

“A total of 1,047 individual cattle serum samples collected in the previous study [8] during 2012–2014 and a follow-up study in 2015 from traditional cattle herds in six districts of four provinces of Zambia (Shibuyunji in Lusaka Province, Mumbwa in Central Province, Mazabuka and Kazungula in Southern Province, and Mwandi and Sesheke in Western Province) were used for the serologic screening (Fig 1 and Table 1).” (lines 96-100).

The authors use an in-house IF method for detection of IgG antibodies to recombinant N protein expressed in HeLa cells. As the authors present the first evidence of CCHFV in Zambia, providing more information on the specificity and sensitivity of the assay using sera from cattle, a publication describing the use of this assay with cattle sera, or confirming the results by detection of anti-glycoprotein antibodies would further strengthening the manuscript.

Thank you for the suggestion. As described in a Method section, the antigen used in an immunofluorescent assay (IFA) in the present study is a mixture of CCHFV nucleoprotein (N)-expressing and non-expressing HeLa cells at a ratio of 1:3. To achieve high specificity, serum samples that showed fluorescent signals uniformly in almost all the cells on a slide were therefore considered as negative irrespective of the signal intensity. This IFA has been already used in serological surveillance carried out in Nigeria for CCHF in domestic ruminants including cattle (Oluwayelu et al. 2020 Emerg Infect Dis). In this previous study, the IFA had 96% concordance with the screening results of an enzyme-linked immunosorbent assay using the recombinant CCHFV N. These explanations were added to the Method (lines 124-127) and Results (lines 194-199) sections in the manuscript with an additional reference #30.

Lines 176-179, 269-276, and 273-274: The authors present and discuss an estimation of the seroprevalence in different districts, can the authors please provide the 95% CI for these estimations.

Thank you for the suggestion. According to the suggestion, 95% confidence intervals of seroprevalence in each district were provided (lines 203-207, 303-306).

The authors have drawn somewhat strong conclusions from the phylogenetic analyses which are based on a limited number of sequences. Can the authors please address these limitations in the Discussion.

Thank you for the comment. We completely agree that there is a limitation to appropriately infer phylogenetic relationship among CCHFVs largely due to limited and geographically biased CCHFV sequence data. This explanation was added to the Discussion as “However, CCHF phylogeography has not been comprehensively understood yet due to limited and geographically biased sequence data of the viruses.” (lines 346-348).

Line 96: Please clarify if the 1047 sera were collected from individual animals or not.

Thank you for the comment. 1,047 serum samples were collected from individual animals. The phrase was corrected as “A total of 1,047 individual cattle serum samples” in line 96.

Lines 180-181: Please clarify if the 526 sera tested by nested RT-PCR was part of the 1047 sera tested for antibodies. In Methods, provide information regarding the treatment and RNA extraction of the sera prior to PCR analysis.

Thank you for the comment. The 526 sera tested by nested RT-PCR was a part of 1,047 cattle sera tested for antibodies. The sentence in lines 180-182 was corrected as follows:

“Because the virus genome was not detected in randomly selected 526 sera out of the aforementioned 1,047 sera by nested reverse transcription PCR (RT-PCR), viremic cattle seemed to be rare.” in line 208-210. In addition, the methodology for RNA extraction was also modified (lines 100-102, 151).

Lines 202-203: Provide the statistical test used and the p-value for determining possible correlation between prevalence and tick species. Also, in Methods add information on which software that was used to perform the analysis.

According to the suggestion, association between CCHFV prevalence and tick species was analyzed by chi-square test using R version 4.0.5. Significant correlation was not observed (p-value = 0.97). The sentence in lines 202-203 was corrected as “No significant correlations between the CCHFV prevalence and tick species were found by chi-square test (p-value = 0.97) (Table 3).” (lines 231-232) In addition, a subsection for the statistical analysis was added in the Methods section (lines 166-168).

Please add information in Methods on the BSL facility and the procedures and conditions used for the attempts to isolate CCHF from tick homogenates (described in Results, line 203).

According to your suggestion, the following paragraph about virus isolation was added in the Methods (lines 140-149).

“**Virus isolation**

As a routine screening of tick-borne viruses, tenfold-diluted tick homogenates were filtrated through 0.45 µm membrane filters (Iwaki) and then inoculated onto Vero E6 (African green monkey kidney) cells. Cells were maintained in Dulbecco's modified Eagle medium supplemented with 2% fetal bovine serum, 2 mM L-glutamine, 4% antibiotic–antimycotic solution (Gibco), and 1.0 mg/mL NaHCO_3_ for 14 days. Blind passages were performed at 14 days post-infection. RNAs were purified from supernatants using the QIAamp viral RNA minikit (Qiagen), and virus growth was examined by RT-PCR. These experiments were performed in the Biosafety level 3 laboratory in Hokudai Center for Zoonosis Control in Zambia, the School of Veterinary Medicine, the University of Zambia.”

Lines 218-219: Although it was not possible to obtain S segment full-length sequences from the other 10 RT-PCR positive ticks, did the authors try to recover partial sequences of the M segment from these ticks to confirm the result?

Thank you for the comment. Actually, we attempted to detect the M genome segments from 11 RNA samples positive for the S genome segments. The M genome segments were not detected from any samples except for ZT15-90. Accordingly, the sentence was modified as follows:

“Partial sequences of the M segment (nucleotide positions 25-554 (535 nt) and 4604-5118 (515 nt)) were also determined from the same tick sample (GenBank accession no. LC534909 and LC534910), while the M segment was not detected from the other 10 samples positive for the S segment. However, the L segment was not detected by RT-PCR with multiple sets of degenerate primers designed based on conserved sequences in CCHFVs (S2 Table)” (lines 250-253).

Line 224-237: Parts of the data presented here are/confirm previous findings from reference 16, please move these parts to the Discussion.

Thank you for the comment. While some data have been already reported in reference #16, the sentences in lines 224-237 explain the results of the phylogenetic analysis independently performed in the present study. Since the data confirming previous findings are also essential to explain the phylogenetic position of CCHFV found in Zambia, we would like to keep these sentences in this paragraph. We appreciate your positive consideration.

Please provide GenBank accession numbers for the sequences used in the phylogenetic analysis. These accession numbers can either be added to the trees or be presented in a table in supplementary linking the names used in the trees with their respective accession number.

Thank you for the comment. The accession numbers of CCHFV genome sequences were presented in S3 Table in the revised manuscript. The sentence “GenBank accession numbers of CCHFV genome sequences used in the phylogenetic analyses are shown in S3 Table.” was added in the Methods (lines 188-189).

**To Reviewer #3**

Authors conclude that CCHFV has moderate circulation throughout Zambia and discuss how their data add to the field's understanding of CCHFV circulation in Africa.

We appreciate your comment.

The manuscript by Kajihara et al investigates the seroprevalence of CCHF in cattle and presence of CCHFV RNA in ticks within Zambia, a country that has not been reported as CCHFV endemic. The manuscript is well written and data well presented. Data adds to our understanding of the circulation of CCHFV within Africa, an understudied region for CCHFV.
Thank you for your positive comment.

Minor comments:

Line 203: Authors state that no infectious virus was recovered from any tick sample, what method was used to attempt to recover infectious virus?

Thank you for the comment. According to your suggestion, the following paragraph about virus isolation was added in the Methods (lines 140-149).

“**Virus isolation**

As a routine screening of tick-borne viruses, tenfold-diluted tick homogenates were filtrated through 0.45 µm membrane filters (Iwaki) and then inoculated onto Vero E6 (African green monkey kidney) cells. Cells were maintained in Dulbecco's modified Eagle medium supplemented with 2% fetal bovine serum, 2 mM L-glutamine, 4% antibiotic–antimycotic solution (Gibco), and 1.0 mg/mL NaHCO_3_ for 14 days. Blind passages were performed at 14 days post-infection. RNAs were purified from supernatants using the QIAamp viral RNA minikit (Qiagen), and virus growth was examined by RT-PCR. These experiments were performed in the Biosafety level 3 laboratory in Hokudai Center for Zoonosis Control in Zambia, the School of Veterinary Medicine, the University of Zambia.”

Table 2: Were ticks collected from individual cattle or were multiple ticks collected from the same cow? Could positive ticks have come from the same cow?

Multiple ticks were collected from each cattle. Therefore, the possibility that positive ticks were collected from the same cow cannot be completely excluded in Chipata, Shibuyunji, and Mumbwa. This possibility was added in the revised manuscript (lines 223-224).

Line 134 - 148: Were no template controls included?
Template controls were not used for the genome screening. Reproducibility of CCHFV genome detection was confirmed by repeated experiments. This information was added in the Methods (lines 164-165).

What is the level of movement of cattle within Zambia? Cattle movement between Zambia and Namibia or South Africa where the Asian M segment viruses have been reported?

Thank you for the comment. In Zambia, more than 80% of the cattle population are maintained in the traditional farmer sector, where livestock is kept by small scale/communal grazing (Mumba et al., 2018 Trop Anim Health Prod). Therefore, long-distance or cross-border cattle transport less frequently takes place. However, transmission of CCHFV or CCHFV-infected ticks via livestock trades cannot be ruled out, as discussed in the manuscript.

Figure 1: Authors should consider adding a symbol such as a star to indicate sampling sites with positive CCHFV tick or cattle samples to make it easier to understand geographically where CCHFV was detected.

Thank you for the comment. According to the suggestion, the areas where CCHFV genome was detected in ticks were indicated by asterisks (*) in Figure 1. Accordingly, the figure legend was also modified (line 110).
